# Supplementary material for: Frequency and laminar profile of feature-specific visual activity revealed by interleaved EEG–fMRI
Source: eLife. 2026 Sep 3;14:RP108408. doi: 10.7554/eLife.108408 (PMC13541303; doi:10.7554/eLife.108408)
Supplement: Peer review history

# Editor report: eLife Assessment

**DOI:** 10.7554/eLife.108408.3.sa0

**Authors:** Peter Kok

**Affiliations:** University College London United Kingdom

**Keywords:** Compelling, Fundamental

This **fundamental** study uses simultaneous EEG and fMRI recordings to shed light on the relationship between alpha and gamma oscillations and specific cortical layers. The sophisticated methodology provides **compelling** evidence for correlations between oscillatory power and the strength and contents of fMRI signals in different cortical layers. This paper will be of interest to neuroscientists studying the role and mechanisms of alpha and gamma oscillations.

# Referee report: Reviewer #1 (Public review)

**DOI:** 10.7554/eLife.108408.3.sa1

In this manuscript, Clausner and colleagues use simultaneous EEG and fMRI recordings to clarify how visual brain rhythms emerge across layers of early visual cortex. They report that gamma activity correlates positively with feature-specific fMRI signals in superficial and deep layers. By contrast, alpha activity generally correlated negatively with fMRI signals, with two a higher frequency within the alpha reflecting feature-specific fMRI signals. This feature-specific alpha code indicates an active role of alpha oscillations in visual feature coding, providing compelling evidence that the functions of alpha oscillations go beyond cortical idling or feature-unspecific suppression.

The study is very interesting and timely. Methodologically, it is state of the art. The findings on a more active role of alpha activity that goes beyond the classical idling or suppression accounts is in line with recent findings and theories. In sum, this paper makes a very nice contribution to the literature. In particular, it provides a novel characterization of how oscillatory signals orchestrate the coding of visual contents in the visual cortex and provides a starting point for further research examining how this oscillatory coding changes across visual contents and tasks.

# Referee report: Reviewer #2 (Public review)

**DOI:** 10.7554/eLife.108408.3.sa2

The authors address a long-standing controversy regarding the functional role of neural oscillations in cortical computations and layer-specific signalling. Several studies have implicated gamma oscillations in bottom-up processing, while lower-frequency oscillations have been associated with top-down signalling. Therefore, the question the authors investigate is both timely and theoretically relevant, contributing to our understanding of feedforward and feedback communication in the brain. This paper presents a novel and complicated data acquisition technique, the application of simultaneous EEG and fMRI, to benefit from both temporal and spatial resolution. A sophisticated data analysis method was executed in order to understand the underlying neural activity during a visual oddball task. The authors defined both feature-specific and feature-unspecific contrasts, further subdivided by EEG power regressors, to examine how orientation information is signalled across cortical layers. Feature specific contrast was established via comparing trials where stimulus orientation (respectively) was left with those where the stimulus orientation was right. Further specifying it depending on EEG power regressors as congruent where stimulus orientation of EEG regressor matches voxel preference or incongruent (stimulus orientation of EEG regressor does not match voxel preference).

Figures are well-designed and appropriately represent the results, which seem to support the overall conclusions. However, some of the claims (particularly those regarding the contribution of gamma oscillations) feel somewhat overstated, as the results offer indeed some significant evidence. On the other hand, the lower-frequency findings are compelling, the functional specificity observed within the alpha frequency band is a particularly interesting result and further highlights the importance of distinguishing feature specificity in order to reveal more nuanced characteristics of neuroimaging data.

Overall, main findings are very interesting, and mainly in line with our current understanding of feedback and feedforward signalling. The paper is well-written, addresses a relevant and timely research question, introduces a novel and elegant analysis approach, and presents interesting findings.

The evidence for gamma involvement in the observed effects is selective: no significant gamma-related clusters were found for the feature-unspecific BOLD signal (Figure 5C,F), with significant effects emerging only in positively responding voxels and only for the contrast between congruent and incongruent conditions in the feature-specific BOLD response. The authors address this in the Discussion, noting that the stimulus may have elicited a weaker gamma response overall, and the contrast of EEG congruent vs. incongruent is necessary in order to achieve the largest contrast-to-noise ratio.

Authors reported negative relationship between the alpha frequency band and the feature specific BOLD signal increases (for congruent condition, Figure 5A,D). Furthermore, testing for the functional specificity between lower vs. upper alpha (Figure 5B,E), the authors included statistical test on the mixed effects model coefficients, and found significant interaction between alpha frequencies in the congruent condition. This interaction was mainly driven by the upper alpha band (which was later confirmed with simple effects analysis) and revealed stronger negative relationship of upper alpha and the BOLD signal for the subtraction of congruent over incongruent conditions. These are exciting results, which further advocate for a more active role of upper alpha band involvement (relative to lower alpha band) in processing visual features.

Expanding on this, the authors have also conducted an exploratory analysis of the relationship between the behavioural findings and underlying neural activity for non-oddball trials (Figure S12 in Supplementary Figures). This confirmed a positive relationship between task performance and alpha frequency, suggesting that high behavioural accuracy is reflected by a stronger modulation of high-frequency alpha power.

This study provides a valuable and exciting contribution to the literature on oscillatory dynamics and laminar fMRI.

Comments on revised version.

Thank you for the thorough revision and for addressing the comments so carefully. The new figures are super beautiful and make the results considerably easier to interpret, they are a real improvement to the paper.

# Author comment: Author response

**DOI:** 10.7554/eLife.108408.3.sa3

**Authors:** Tommy Clausner, Jose JP Marques, Rene Scheeringa, Mathilde Bonnefond

**Affiliations:** Inserm Lyon France; Donders Institute for Brain, Cognition and Behaviour Nijmegen Netherlands; University of Duisburg-Essen Essen Germany; Inserm Lyon France

The following is the authors’ response to the original reviews.

**Public Reviews:**

**Reviewer #1 (Public review):**

In this manuscript, Clausner and colleagues use simultaneous EEG and fMRI recordings to clarify how visual brain rhythms emerge across layers of early visual cortex. They report that gamma activity correlates positively with feature-specific fMRI signals in superficial and deep layers. By contrast, alpha activity generally correlated negatively with fMRI signals, with two higher frequencies within the alpha reflecting feature-specific fMRI signals. This feature-specific alpha code indicates an active role of alpha oscillations in visual feature coding, providing compelling evidence that the functions of alpha oscillations go beyond cortical idling or feature-unspecific suppression.

The study is very interesting and timely. Methodologically, it is state-of-the-art. The findings on a more active role of alpha activity that goes beyond the classical idling or suppression accounts are in line with recent findings and theories. In sum, this paper makes a very nice contribution. I still have a few comments that I outline below, regarding the data visualization, some methodological aspects, and a couple of theoretical points.

The authors put a lot of effort into the figure design. For instance, I really like Figure 1, which conveys a lot of information in a nice way. Figures 3 and 4, however, seem over engineered, and it takes a lot of time to distill the contents from them. The fact that they have a supplementary figure explaining the composition of these figures already indicates that the authors realized this is not particularly intuitive. First of all, the ordering of the conditions is not really intuitive. Second, the indication of significance through saturation does not really work; I have a hard time discerning the more and less saturated colors. And finally, the white dots do not really help either. I don't fully understand why they are placed where they are placed (e.g., in Figure 3). My suggestion would be to get rid of one of the factors (I think the voxel selection threshold could go: the authors could run with one of the stricter ones, and the rest could go into the supplement?) and then turn this into a few line plots. That would be so much easier to digest.

We thank the reviewer for their insightful comments. Below we will address each point separately and highlight the changes made to the manuscript. In agreement with the reviewer we have recompiled Figures 4 and 5 (previously Figures 3 and 4). The new figures only present results for the 10% voxel selection threshold (with 5% and 25% moved to Supplementary Figures, see Figures S1-S9). Instead of the radially arranged layout, we opted for a more traditional figure layout, which significantly improved readability.

(2) The division between high- and low-frequency alpha in the feature-specific signal correspondence is very interesting. I am wondering whether there is an opposite effect in the feature-unspecific signal correspondence. Would the high-frequency alpha show less of a feature-unspecific correlation with the BOLD?

Following the reviewer’s interesting suggestion, we added the low/high frequency alpha analysis to the feature-unspecific analysis. Indeed, we have found a significant interaction between the sign of the signal change for selected voxel (positive vs. negative BOLD) and alpha sub-band (low vs high frequency alpha). An analysis of simple effects did not reveal any significant effects, however we found a trend level difference (p=0.097) between low and high-frequency alpha for the positive voxel sub-selection. This indicates a stronger negative relationship between upper alpha and the positive BOLD signal as compared to lower alpha. We interpret this result as partial evidence for a feature-related contribution of the upper alpha band. “Active” cortical patches (positive BOLD) are most likely involved in the processing of visual features (irrespective of the specific feature), and additionally a more general (possibly attention-related) activation. In turn the negative BOLD signal might contain less feature-specific activation and is most likely related to attention-driven deactivation. The negative relationship between alpha and negative BOLD could thus be interpreted as an indirect effect, resulting from a reduced alpha decrease in cortical patches responding to non-attended receptive field locations. However, the involvement of attention-related processes remains speculative, since attention was not explicitly manipulated as part of the experiment.

We have added Figure 4 B.

We have also added this section to the Results:

“We furthermore found a significant interaction (p_FDR_<0.05) between positive or negative BOLD signal change and lower or upper α sub-bands (8 - 10 or 11 - 13 Hz respectively) by means of a linear mixed effects model. An analysis of simple effects revealed that upper α frequencies are stronger negatively related to the positive BOLD signal as compared to lower α on a trend level (p_FDR_ = 0.097).”

And the following section of the Discussion was extended:

“The significant interaction between the sign of the BOLD signal deflection and upper or lower α bands (see Figure 4 B) further indicates that multiple α-related processes contribute differentially to positive or negative BOLD. "Active" cortical patches (positive BOLD) are most likely involved in the processing of visual features (irrespective of the specific feature), and additionally a more general (possibly attention-related) activation. In turn the negative BOLD signal might contain less feature-specific activation and is most likely related to attention-driven deactivation. This hypothesis receives additional support from the trend-level difference in α sub-bands for positive BOLD, indicating that lower α is less related to the active, possibly feature-related processes. The absence of this difference for negative BOLD again indicates a broader, more general process. Future experiments manipulating visual features and attention might reveal a differential upper and lower α response to attended visual features and a more general relationship between α (and possibly superficial layer cortical activity) for suppressed (unattended) receptive fields.”

(3) In the discussion (line 330 onwards), the authors mention that low-frequency alpha is predominantly related to superficial layers, referencing Figure 4A. I have a hard time appreciating this pattern there. Can the authors provide some more information on where to look?

We thank the reviewer for pointing out the lack of clarity of this section in the Discussion. We have now rephrased the Discussion, focusing more on the laminar difference and keeping the frequency difference to a separate paragraph. Our main argument for possibly multiple alpha-related processes are twofold: a difference in alpha frequency depending on the underlying analysis (low vs high frequency alpha) and a different layer distribution (superficial layers vs. superficial and deep layers, depending on the analysis). The respective section in the Discussion now focuses on the laminar difference only. We find a negative relationship between alpha and the BOLD signal most prominently in superficial layers (feature-unspecific contrast for the BOLD signal with negative t-values; Figure 4). In addition, we find a superficial and deep layer contribution for the feature-specific contrast (congruent - incongruent; Figure 5A). While the here presented experiment was set out to investigate feature-specific processes, the meaning of the feature-unspecific results are of speculative nature. Future experiments should target the laminar difference between feature-specific and unspecific processes with respect to alpha frequency and layer distribution directly.

We have modified the respective sections in the Discussion:

“Furthermore, we observed that the relationship between the feature-specific BOLD signal and α is predominantly linked to frequencies above 11 Hz (see Figure 5A). An analysis of upper and lower α sub-bands revealed a significant interaction between congruence condition and α frequency. This interaction was mainly driven by the upper α band (11 to 13 Hz). For congruently selected voxels, the negative relationship was significantly stronger (over lower α), while for incongruent selection it was significantly weaker. No such difference has been observed for the lower α component, which indicates a more feature-specific involvement of upper α and a more general modulatory effect for lower α frequencies. Since individual frequency variations have been included as a random slope in the linear mixed-effects model, these effects cannot be explained by a subset of participants driving lower or upper α separately. Specifically our findings on upper α indicate that α is not exclusively linked to global signal modulations, which has been the traditional perspective [...]”

“Not only did we find a dissociation in the frequency domain between the relationship of α and the BOLD signal, but furthermore found that the laminar activation patterns provide further evidence for potentially multiple α-related processes. The association between α and the BOLD signal was strongest in superficial layers for negative BOLD activity and feature-specific activity (see Figure 4A and 5A). However, deep layer-related α effects were limited to feature-specific processes only (see Figure 5 A Co-Inco). These findings suggest that superficial layer α reflects are broader, more general process, while deep layer α operates more narrowly, linked to the processing of the visual features themselves. Previous findings using laminar fMRI (which did not include the investigation of oscillatory activity), indicate that superficial layer activity might be more related to the modulation of attention [...]”

(4) How did the authors deal with the signal-to-noise ratio (SNR) across layers, where the presence of larger drain veins typically increases BOLD (and thereby SNR) in superficial layers? This may explain the pattern of feature-unspecific effects in the alpha (Figure 3). Can the authors perform some type of SNR estimate (e.g., split-half reliability of voxel activations or similar) across layers to check whether SNR plays a role in this general pattern?

We agree with the reviewer that the vascular draining effect typically leads to increased signal change in superficial layers, the effect on (t)SNR however might be less straightforward. We did not include any counteracting measures, because we were not interested in the amplitude of the signal change, but now include an estimate of tSNR (See Figure S10 in Supplementary Figures). We found that in fact the signal-to-noise ratio is higher in deep layers. Most importantly however, the tSNR layer profiles we identified do not reflect the correlation layer result patterns of the combined EEG-fMRI analysis. This indicates that our results are most likely not the result of tSNR differences. In order to confirm our tSNR pattern we have also conducted a second layer analysis based on the LAYNII toolbox, which assigns voxels between pial and white matter to distinct layers (as compared to our fraction-based approach) and found a similar profile as with our initial analysis. However, absolute tSNR values were found to be higher for our weighted layer analysis. We speculate that while functionally relevant components of the BOLD signal drain towards superficial layers, physiological noise components will drain towards superficial layers as well.

It is furthermore worth pointing out that for the contrast (congruent - incongruent), the vascular draining effect would cancel out between the conditions. Our findings on superficial and deep layers for those contrasts can hence not be explained by vascular draining at all.

We have added Figure S10 to Supplementary Figures and the following section to the Discussion:

“A major concern for laminar fMRI is the vascular draining effect (Markuerkiaga et al., 2016), which typically leads to increased signal amplitudes closer to the surface. Here, we did not investigate the signal change per se, but rather the relationship with EEG power changes. To ensure that the results do not stem from differences in tSNR across layers, we conducted a tSNR analysis (see Figure S10 in Supplementary Figures). We found that the highest tSNR was obtained from deep layers, as compared to middle and superficial layers. To verify, we computed the tSNR using a second layering algorithm (LayNii, see Huber et al. 2021), which yielded lower absolute values, but a comparable layer profile. The obtained tSNR is not reflected in any of our result profiles (see Figures 4 and 5), which strengthens the validity of the here presented results. We speculate that tSNR in deep layers is higher, because both functionally relevant components of the BOLD signal and physiological noise components drain towards superficial layers.”

(5) The GLM used for modelling the fMRI data included lots of regressors, and the scanning was intermittent. How much data was available in the end for sensibly estimating the baseline? This was not really clear to me from the methods (or I might have missed it). This seems relevant here, as the sign of the beta estimates plays a major role in interpreting the results here.

This is a very important remark and we would like to apologise for the confusion. It was not clear in the manuscript that the GLM was computed on z-transformed fMRI data. We have not specifically collected any “baseline volumes”. A positive beta value would indicate that the sign of the predictor matches the sign of the BOLD signal deflection (and vice versa).

We have added or modified the following sections in Results and Methods respectively:

“Before the GLM was computed, the fMRI data was z-transformed across time, separately for each block and voxel.”

“A general linear model (GLM) has been computed with predictors for each TF bin separately for all voxels in V1 that later have been sub-selected according to the respective condition. Time courses for each voxel have been z-transformed before the GLM was computed for each voxel and experimental block separately. Afterwards, each of the resulting regression coefficients (β values) were multiplied with the voxel-specific layer weights that have been obtained as described above.”

(6) Some recent research suggests that gamma activity, much in contrast to the prevailing view of the mechanism for feedforward information propagation, relates to the feedback process (e.g., Vinck et al., 2025, TiCS). This view kind of fits with the localization of gamma to the deep layer here?

(7) Another recent review (Stecher et al., 2025, TiNS) discusses feature-specific codes in visual alpha rhythms quite a bit, and it might be worth discussing how your results align with the results reported there.

We would like to thank the reviewer for pointing out these papers. Yes, we believe that those could be very related to the effects reported here. At the time of writing the initial manuscript we were not aware of the mentioned publications.

We have now included these papers in the Discussion:

“Recent publications on the information exchange within and between primary visual cortex areas of macaques also reported deep layer γ band activity depending on the stimulus material (Gieselmann et al., 2022; Ferro et al., 2021). Those publications challenge the feed-forward exclusivity of γ altogether by revealing intra-area feedback communication in V1 from layer 5 to layer 6 and layer 6 to supra-granular layers. Possibly, the relationship between γ and deep layer BOLD we observed is also related to similar processes (Vinck et al., 2025).”

“Similarly, in a recent opinion article, Stecher et al. (2025) promote the idea of "content-aware" α-oscillations. In agreement with our results, the authors argue that α-oscillations are related to content-specific feedback signals, reflected in increased decoding performance based on α power of top-down related processes, even prior to the onset of the stimulus (Hetenyi et al., 2025).. Accordingly, we interpret the lower α effect [...]”

**Reviewer #2 (Public review):**

The authors address a long-standing controversy regarding the functional role of neural oscillations in cortical computations and layer-specific signalling. Several studies have implicated gamma oscillations in bottom-up processing, while lower-frequency oscillations have been associated with top-down signalling. Therefore, the question the authors investigate is both timely and theoretically relevant, contributing to our understanding of feedforward and feedback communication in the brain. This paper presents a novel and complicated data acquisition technique, the application of simultaneous EEG and fMRI, to benefit from both temporal and spatial resolution. A sophisticated data analysis method was executed in order to understand the underlying neural activity during a visual oddball task. Figures are well-designed and appropriately represent the results, which seem to support the overall conclusions. However, some of the claims (particularly those regarding the contribution of gamma oscillations) feel somewhat overstated, as the results offer indeed some significant evidence, but most seem more like a suggestive trend. Nonetheless, the paper is well-written, addresses a relevant and timely research question, introduces a novel and elegant analysis approach, and presents interesting findings. Further investigation will be important to strengthen and expand upon these insights.

One of the main strengths of the paper lies in the use of a well-established and straightforward experimental paradigm (the visual oddball task). As a result, the behavioural effects reported were largely expected and reassuring to see replicated. The acquisition technique used is very novel, and while this may introduce challenges for data analysis, the authors appear to have addressed these appropriately.

Later findings are very interesting, and mainly in line with our current understanding of feedback and feedforward signalling. However, the layer weight calculation is lacking in the manuscript. While it is discussed in the methods, it would help to briefly explain in the results how these weights are calculated, so that the reader can better follow what is being interpreted.

Line 104 states there is one virtual channel per hemisphere for low and high frequencies. It may be helpful to include the number of channels (n=4) in the results section, as specified in the methods. Also, this raises the question of whether a single virtual channel (i.e., voxel) provides sufficient information for reproducibility.

We thank the reviewer for encouraging us to clarify the virtual channel selection and we agree that the current description could be misleading. Indeed, we selected 4 virtual channels in total: 1 for each frequency band (alpha/gamma), for each hemisphere separately. The main goal of this selection was to find the clearest response of that frequency band to the task. Previous publications used a supervised (ICA-based) approach to extract those responses. To increase reproducibility, we have chosen an unsupervised beamformer-based approach. The reconstruction of time or frequency-resolved sources in the brain typically yields spatially highly correlated results. Publications focusing on this type of analyses report a spatial extent of typically multiple centimetres, which here is the case as well (see Figure 3A of the updated manuscript). As such, the single voxel selection boils down to selecting the peak response within a large patch of very similarly responding voxels. Using this approach we were able to select the frequency response with the highest possible SNR. We do not however claim that the respective single voxel is exclusively carrying this information. In addition we have added a short explanation to the Discussion, since we believe that virtual channel selection with a different objective (e.g. maximising the difference between conditions or maximising cross-frequency coupling, etc.) could indeed profoundly impact the EEG-fMRI correlation, which would open up opportunities for interesting analyses that are however beyond the scope of this project.

We have added the following section to the Discussion:

“Future work might also vary the exact virtual channel selection for obtaining EEG-based regressors. Here, we focused on the grid points (voxel locations) with the strongest α or γ response for each frequency band in each hemisphere, derived from the average frequency response to maximise SNR. However, selecting the respective virtual channels based on the response to specific stimulus features or the interaction between high and low frequency bands are possibilities worth exploring in future work.”

One area that would benefit from further clarification is the interpretation of gamma oscillations. The evidence for gamma involvement in the observed effects appears somewhat limited. For example, no significant gamma-related clusters were found for the feature-unspecific BOLD signal (Figure 2). Significant effects emerged only when the analysis was restricted to positively responding voxels, and even then, only for the contrast between EEG-coherent and EEG-incoherent conditions in the feature-specific BOLD response. It remains unclear how to interpret this selective emergence of gamma-related effects. Given previous literature linking gamma to feedforward processing, one might expect more robust involvement in broader, feature-unspecific contrasts. The current discussion presents the gamma-related findings with some confidence, and the manuscript would benefit from a more nuanced reflection on why these effects may not have appeared more broadly. The explanation provided in line 230, that restricting the analysis to positively responding voxels may have increased the SNR, is reasonable, but it may not fully account for the absence of gamma effects in V1's feature-unspecific response. Including the actual beta values from Figure 4 in the legend or main text would also help readers better assess the strength and specificity of the reported effects.

We agree with the reviewer that the missing gamma-band response for the feature-unspecific signal, as well as the limitation of the effect solely to the feature-specific contrast for positive voxel selections only was unexpected. In fact, based on previous literature, we were expecting a feature-unspecific effect in the gamma band as well. However, the literature on laminar level EEG-fMRI is sparse and previous experiments used tasks that did not allow for the separation into distinct features (here left or right-oriented gratings). While we cannot fully explain the absence of the gamma effect for the feature-unspecific condition, we reasoned that our stimuli evoked weaker gamma band responses compared to previous literature.

The fact that we only see a significant gamma band response for the contrast for positive voxel selections can be interpreted twofold: First, previous experiments limit their analyses to positive BOLD responses only, for which we find an effect as well. Second, the fact that a significant effect could only be obtained for the contrast, might indicate that gamma band activity is related to the actual features themselves. A cortical column responding to left-oriented gratings would then be related to a gamma band response linked to that orientation. If this response to a single orientation could not be fully captured due to SNR-related issues, we would not see this effect in the congruent-only condition and also not in the feature-unspecific condition (because this boils down to both congruent conditions combined). If gamma-band oscillations are actually reflecting the response of a column to a certain orientation, then the lowest possible response would be found for the exact orthogonal orientation (here the incongruent condition). The contrast between most preferred and most not-preferred orientation might have helped to overcome the inherently low SNR, explaining the results for the contrast.

Lastly, we did not include actual beta values in the main text, because those might be misleading. We compute the relationship between EEG power and the BOLD signal for every voxel separately, then weighted the result with the respective layer weight and lastly aggregated across voxels.This means that the beta values express the strength of the association between EEG and fMRI for an average voxel. For this reason the values are tiny and the values themselves are less meaningful than “typical” beta values.

We have added or modified the following sections in the Discussion or Methods respectively:

“Based on previous literature, we expected a γ band effect for the congruent condition of the feature-specific analysis (Scheeringa et al., 2016), which we did not observe. A possible explanation could be the used stimulus material in our experiment as compared to Scheeringa et al., (2016). Muthukumaraswamy et al., (2013) found that stationary gratings evoke a weaker γ band response as compared to moving annular stimuli that have been used by Scheeringa and colleagues. If γ is related to the processing of the actual features themselves (e.g. to a column preferably responding to left-oriented gratings), then contrasting congruent and incongruent voxel selections provides the largest possible contrast-to-noise ratio (CNR). In turn annular stimuli as previously used might have activated all possible orientations and thus might have greatly boosted γ SNR.”

“The described procedure of computing a GLM based on z-transformed data using z-transformed predictors yields β-coefficients that reflect the average relationship of a single voxel's BOLD response for a given layer (fraction of the single voxel's β) with EEG power changes of a specified frequency.”

Relating to behavioural findings for underlying neural activity, could the authors test on a trial-by-trial basis how behavioural performance relates to the BOLD signal / oscillatory activity change? Line 305 states that "Since behavioural performance in the present study was consistently high at 94% on average and participants were instructed to respond quickly to potential oddball stimuli, a higher alpha frequency might reflect a more successful stimulus encoding and hence faster and more accurate behavioural performance." Also, this might help to relate the findings to the lower vs upper alpha functionality difference.

This is a very interesting suggestion. We now include an exploratory analysis of the relationship between frequency and behavioural performance in the Supplementary Figures (see Figure S12). We did not perform a correlation between behavioural performance and alpha over trials because of the low numbers of oddball trials (N=40) and very limited number of false responses (94% response accuracy on average). However, we computed a correlation across participants. After averaging the alpha time-frequency spectrum across non-oddball trials, the individual alpha frequency was determined by the frequency where the alpha decrease (between 0.1 and 0.8 s post-stimulus) was largest. The correlation between alpha frequency and either reaction times and d’ (as a measure for accuracy), yields a significantly positive relationship between d’ and alpha frequency. This indicates that alpha frequency is related to task performance. We interpret those exploratory findings such that high behavioural accuracy is reflected by a stronger modulation of high-frequency alpha power.

We have added Figure S12 to Supplementary Figures.

We have also added the following sections to Results and Discussion respectively:

“An exploratory analysis of the relationship between individual α frequency (IAF) and task performances underlines this finding (see Figure S12 in Supplementary Figures). Thereby the IAF was obtained from the average α power spectrum of each participant. The frequency with the strongest decrease between 0.1 and 0.8 s after stimulus onset served as the IAF. We correlated IAF with average response times to correct oddball trials and d' as a measure for accuracy and found a significant positive correlation between IAF and d' (p < 0.05).”

“We exploratively correlated the average IAF during non-oddball trials with the average task accuracy (d') across participants and indeed found IAF and task performance to be positively correlated (See Figure S12 in Supplementary Figures).”

In Figure 4, the EEG alpha specificity plot shows relatively large error bars, and there is visible overlap between the lower and upper alpha in both congruent and incongruent conditions. While upper alpha shows a positive slope across conditions and lower alpha remains flat, the interaction appears to be driven by the change from congruent to incongruent in upper alpha. It is worth clarifying whether the simple effects (e.g., lower vs upper within each condition) were tested, given the visual similarity at the incongruent condition. Overall, the significant interaction (p < 0.001, FDR-corrected) is consistent with diverging trends, but a breakdown of simple effects would help interpret the result more clearly. Was there a significant difference between lower and upper alpha in congruent or incongruent conditions?

We thank the reviewer for this important remark and have added a simple effects analysis (see Figures 4 b and 5 b, e). We found that the main driver for the interaction between congruence condition and alpha frequency is upper alpha. Specifically the negative relationship between upper alpha and the BOLD signal is significantly stronger for the congruent condition and weaker for the incongruent condition. This indicates the upper alpha indeed is related to the processing of visual features.

We have added a simple effects analysis (See Figures 4 and 5).

We have added or modified the following in Results, Discussion and Methods respectively:

In Results:

“We furthermore found a significant interaction (p_FDR_ < 0.05) between positive or negative BOLD signal change and lower or upper α sub-bands (8 - 10 or 11 - 13 Hz respectively) by means of a linear mixed effects model. An analysis of simple effects revealed that upper α frequencies are stronger negatively related to the positive BOLD signal as compared to lower α on a trend level (p_FDR_ = 0.097).”

“After correcting for multiple comparisons, we found a significant interaction (p_FDR_ < 0.001). This interaction is mainly driven by the upper α sub-band, as indicated by the simple effects analysis. We found a significantly stronger negative relationship of upper α and the BOLD signal for congruent selections (p_FDR_ < 0.01) and the reverse for the incongruent condition (p_FDR_ < 0.01), as well as a significantly stronger negative relationship within the upper α sub-band for congruent over incongruent voxel selections (p_FDR_ < 0.01).”

“This interaction is mainly driven by the upper α sub-band, as indicated by the simple effects analysis, which revealed a significantly stronger negative relationship of upper α and the BOLD signal for congruent over incongruent selections (p_FDR_ < 0.001).”

In Discussion:

“An analysis of upper and lower α sub-bands revealed a significant interaction between congruence condition and α frequency. This interaction was mainly driven by the upper α band (11 to 13 Hz). For congruently selected voxels, the negative relationship was significantly stronger (over lower α), while for incongruent selection it was significantly weaker. No such difference has been observed for the lower α component, which indicates a more feature-specific involvement of upper α and a more general modulatory effect for lower α frequencies.”

In Methods:

“Significant interactions were decomposed into simple effects using Wald tests on the model coefficients, ensuring that post-hoc comparisons were derived from the same statistical global variance as the primary interaction.”

Overall, this study provides a valuable contribution to the literature on oscillatory dynamics and laminar fMRI, though some interpretations would benefit from further clarification or qualification.

**Reviewer #3 (Public review):**

Summary:

Clausner et al. investigate the relationship between cortical oscillations in the alpha and gamma bands and the feature-specific and feature-unspecific BOLD signals across cortical layers. Using a well-designed stimulus and GLM, they show a method by which different BOLD signals can be differentiated and investigated alongside multiple cortical oscillatory frequencies. In addition to the previously reported positive relationship between gamma and BOLD signals in superficial layers, they show a relationship between gamma and feature-specific BOLD in the deeper layers. Alpha-band power is shown to have a negative relationship with the negative BOLD response for both feature-specific and feature-unspecific contrasts. When separated into lower (8-10Hz) and upper (11-13Hz) alpha oscillations, they show that higher frequency alpha showed a significantly stronger negative relationship with congruency, and can therefore be interpreted as more feature-specific than lower frequency alpha.

Strengths:

The use of interleaved EEG-fMRI has provided a rich dataset that can be used to evaluate the relationship of cortical layer BOLD signals with multiple EEG frequencies. The EEG data were of sufficient quality to see the modulation of both alpha-band and gamma-band oscillations in the group mean VE-channel TFS. The good EEG data quality is backed up with a highly technical analysis pipeline that ultimately enables the interpretation of the cortical layer relationship of the BOLD signal with a range of frequencies in the alpha and gamma bands. The stimulus design allowed for the generation of multiple contrasts for the BOLD signal and the alpha/gamma oscillations in the GLM analysis. Feature-specific and unspecific BOLD contrasts are used with congruently or incongruently selected EEG power regressors to delineate between local and global alpha modulations. A transparent approach is used for the selection of voxels contributing to the final layer profiles, for which statistical analysis is comprehensive but uses an alternative statistical test, which I have not seen in previous layer-fMRI literature.

A significant negative relationship between alpha-band power and the BOLD signal was seen in congruently (EEGco) selected voxels (predominantly in superficial layers) and in feature-contrast (EEGco-inco) selected (superficial and deep layers). When separated into lower (8-10Hz) and upper (11-13Hz) alpha oscillations, they show that higher frequency alpha showed a significantly stronger negative relationship with congruency than lower frequency alpha. This is interpreted as a frequency dissociation in the alpha-BOLD relationship, with upper frequency alpha being feature-specific and lower frequency alpha corresponding to general modulation. These results are a valuable addition to the current literature and improve our current understanding of the role of cortical alpha oscillations.

There is not much work in the literature on the relationship between alpha power and the negative BOLD response (NBR), so the data provided here are particularly valuable. The negative relationship between the NBR and alpha power shown here suggests that there is a reduction in alpha power, linked to locally reduced BOLD activity, which is in line with the previously hypothesized inhibitory nature of alpha.

Weaknesses:

It is not entirely clear how the draining vein effect seen in GE-BOLD layer-fMRI data has been accounted for in the analysis. For the contrast of congruent-incongruent, it is assumed that the underlying draining effect will be the same for both conditions, and so should be cancelled out. However, for the other contrasts, it is unclear how the final layer profiles aren't confounded by the bias in BOLD signal towards the superficial layers. Many of the profiles in Figure 3 and Figure 4A show an increased negative correlation between alpha power and the BOLD signal towards the superficial layers.

We thank the reviewer for this important remark. Reviewer 1 raised a similar concern and I would like to refer you to our response to Reviewer 1, point 4. The veinal draining typically results in a higher signal change closer to the cortical surface. We did not take any measures to counteract this effect, but provide an analysis of tSNR in Supplementary Figures (see Figure S10). Possibly due to the drainage of physiological noise towards the surface, we found the highest tSNR in deep, followed by middle and superficial layers. To verify those results we computed the same analysis using a second layering algorithm, which resulted in the same profile, but overall less tSNR. Crucially the tSNR profile is not reflected in our EEG-fMRI results.

We have added Figure S10 to Supplementary Figures and the following section to the Discussion:

“A major concern for laminar fMRI is the vascular draining effect (Markuerkiaga et al., 2016), which typically leads to increased signal amplitudes closer to the surface. Here, we did not investigate the signal change per se, but rather the relationship with EEG power changes. To ensure that the results do not stem from differences in tSNR across layers, we conducted a tSNR analysis (see Figure S10 in Supplementary Figures). We found that the highest tSNR was obtained from deep layers, as compared to middle and superficial layers. To verify, we computed the tSNR using a second layering algorithm (LayNii, see Huber et al. 2021), which yielded lower absolute values, but a comparable layer profile. The obtained tSNR is not reflected in any of our result profiles (see Figures 4 and 5), which strengthens the validity of the here presented results. We speculate that tSNR in deep layers is higher, because both functionally relevant components of the BOLD signal and physiological noise components drain towards superficial layers.”

When investigating if high alpha (8-10 Hz) and low alpha (11-13 Hz) are two different sources of alpha, it would be beneficial to show if this effect is only seen at the group level or can be seen in any single subjects. Inter-subject variability in peak alpha power could result in some subjects having a single low alpha peak and some a single high alpha peak rather than two peaks from different sources.

We agree with the reviewer that a bias in a subset of participants to generally higher or lower alpha frequencies could potentially skew the presented results. While the initially computed model included a random intercept for the frequencies, we have now added the random slope as well. This ensures that the difference between low and high frequency alpha is indeed only driven by the difference in condition and not the result of individual differences across conditions themselves.

In order to verify that not a small subset of participants is driving the result pattern, we also computed the fraction of participants that either show the dual alpha pattern (i.e. follow the exact pattern of the group average), contribute to the group average with a single peak or contradict the pattern entirely. Thereby, 40.4% of all participants show a dual alpha pattern, 38.4% a single alpha pattern in the direction of the group average and 21.2% contradict the group average. See Author response image 1:

**Author response image 1.** Alpha Response Patterns with Example Subjects: V1 Feature Specific Contrast.

We would also like to highlight our added exploratory analysis of the relationship between alpha frequency and behavioural performance, which was requested by Reviewer 2, point 3. We find a significant positive correlation between alpha frequency and task performance on a group level. This indicates that higher alpha frequencies might be related to better discrimination of visual features. We speculate that participants with better task performance are capable of modulating their upper alpha more than participants with worse performance.

We have added Figure S12 to Supplementary Figures.

We have also added the following sections to Results and Discussion respectively:

“An exploratory analysis of the relationship between individual α frequency (IAF) and task performances underlines this finding (see Figure S12 in Supplementary Figures). Thereby the IAF was obtained from the average α power spectrum of each participant. The frequency with the strongest decrease between 0.1 and 0.8 s after stimulus onset served as the IAF. We correlated IAF with average response times to correct oddball trials and d' as a measure for accuracy and found a significant positive correlation between IAF and d' (p < 0.05).”

“We exploratively correlated the average IAF during non-oddball trials with the average task accuracy (d') across participants and indeed found IAF and task performance to be positively correlated (See Figure S12 in Supplementary Figures).”

The figure layout used to present the main findings throughout is an innovative way to present so much information, but it is difficult to decipher the main findings described in the text. The readability would be improved if the example (Appendix 0 - Figure 1) in the supplementary material is included as a second panel inside Figure 3, or, if this is not possible, the example (Appendix 0 - Figure 1) should be clearly referred to in the figure caption.

Since Reviewer 1 suggested using an entirely different figure layout, we now opted to remove some information from the main text figures (we only show the 10% threshold, but 5% and 25% is in Supplementary Figures) and chose a more common figure layout. See Figures 4 and 5.

**Recommendations for authors:**

**Reviewer #2 (Recommendations for the authors):**

The contrasts used in the analysis are not clearly introduced in the main text. While the methods section explains them more thoroughly, some of this explanation would be better placed in the results section, where the contrasts are first used. Specifically, the concepts of "feature-specific" vs. "feature-unspecific" BOLD signals are introduced with a very brief definition, which could be confusing for readers. The same applies to the terms EEG co and EEG inco; it would help to briefly explain these when they are first mentioned in the results. The supplementary figures and legends are helpful, so it is clear that the authors were prioritising clarity overall.

The respective analyses are now also explained in the Results section:

“During each trial either a left or a right-oriented grating was presented, from which two types of analyses have been derived: feature-unspecific BOLD activation (i.e. the response to any stimulus orientation), and feature-specific BOLD activation (i.e. the response to a specific stimulus orientation or the contrast between them). Thereby, fMRI data and EEG-based regressors could either be combined congruently (Co) by combining the BOLD signal of orientation-selective voxels with EEG-based regressors built from the same orientation trials, or incongruently (Inco), by combining the orientation-specific BOLD signal with EEG-based regressors built from the other orientation trials. Finally, those two congruency conditions have been contrasted (Co-Inco).”

Figures are overall clear and illustrative of the results. For Figure 4, however, the use of dotted elements makes it somewhat harder to interpret what's being shown. While the supplementary figure clarifies the findings, rephrasing the figure legend to explain what the dotted lines represent would be helpful.

Figures 4 and 5 have been replaced with a new layout and legends have been improved.

The reported ranges overlap (e.g., alpha: 2-32 Hz; gamma: 20-120 Hz). It would be helpful to explain why such overlapping bands were chosen.

Both frequency bands of interest differ slightly in their later time-frequency analysis (i.e. number of tapers and filter type). The overlap itself is not meaningful per se and results from the selection of a wide band for each respective sub-band. This wide selection was chosen to avoid filter artefacts. For the alpha sub-band, we also wanted to ensure that the beta spectrum is covered which also includes the alpha harmonic and for the gamma band that the full range of high-frequency activity is captured (e.g. EMG activity).

Only a single time point was used for baseline correction of the low alpha band. Is this typical? The authors note that due to the gradient artefact arising in the pre-stimulus period, the baseline correction is somewhat difficult, although further clarification would be useful here.

Relatedly, was pilot scanning conducted? If so, was the presence of strong gradient artefacts unexpected? More details about this would strengthen the methodological transparency.

Indeed only a single time bin was used as the baseline for the alpha sub-band. After the piloting phase a slight adjustment to the final fMRI sequence has been made which was not expected to introduce gradient artefacts so close to the onset of the stimulus. Unexpectedly, those artefacts were visible until 300 ms before the onset of the stimulus. Similarly, a pre-stimulus alpha was observed (starting 250 ms before the onset of the stimulus), which we also aimed to exclude from the baseline period. In the end only the time bin centered at 300 ms prior to stimulus onset was chosen. However, this time bin contains 400 ms of data (the width of the window for the time frequency analysis). Thus, the term time point was misleading, because the actual time window that made up the baseline is 500 ms to 100 ms prior to the onset of the stimulus.

We have adjusted our wording in Methods to make this more clear:

“For this reason, the low frequency baseline period comprised only a single 400 ms time bin centred around -0.3 s, because a pre-stimulus α decrease was expected starting around 0.25 s prior to stimulus onset.”

Including a one-sentence explanation of the AROS test in the main text for clarity. As line 796 in the methods: "Each significant cluster has been further processed by means of an auto-regressive rank order similarity (aros) test (Clausner and Gentili, 2022). The fundamental idea behind the AROS test is whether group averages (i.e. averages of the signal of cortical layer in the present case), can be ranked such that the rank order is explained significantly better by the data than it would if the average data could not be meaningfully sorted (i.e. is shuffled)."

An explanation has been added to the Results section:

“Each significant cluster was then averaged along the frequency dimension at the widest point to enable an auto-regressive rank order similarity (aros) test Clausner & Gentili (2022), testing the laminar activation profile. The aros test transforms the layer averages into a rank order and tests - using a permutation procedure - if the rank order of the layer averages explains the data better than a random rank order (shuffled layer labels) would.”

Line 223: "In fact, an analysis of the relationship between the EEG signal and the BOLD signal that focused on the feature contrast only (L - R; independent of the comparison to baseline) revealed a trend-level result with an even stronger deep layer contribution as compared to superficial layers." Could you point to which figure represents this finding - Figure 4B?

This refers to Figure 4A in the old manuscript, for the 25% threshold for the gamma band. Since now the new figures do not include the 25% threshold anymore, it refers to Figure S4i.

The number of participants is missing from the main text. Including this in the results section would improve clarity.

The description of our sample has been moved from Methods to Results.

Given the complexity of the data acquisition and analysis, the well-designed and easy-to-follow analysis pipeline figure (currently in the supplement) would be better placed in the main text.

The mentioned Figure has been moved to the main text (now Figure 2).

Also, simply out of curiosity, what do the authors think about the theta blob around 200ms post-stimulus?

The theta blob most likely reflects the post-stimulus ERP as often observed in response to visual stimuli. We hypothesise that it is stronger in the middle and superficial layers, but we did not want to extend too much the scope of this paper. Additional analyses could be performed in the future on this evoked activity.

**Reviewer #3 (Recommendations for the authors):**

(1) Minor Corrections to the text and figures:

We would like to thank the reviewer for the very valuable recommendations. Below we shortly describe how each suggestion has been implemented.

We have made the white box more clear (see Figure 3 B).

(b) Page 10: Top of 2nd paragraph - 'The full experimental protocol comprised a high resolution anatomical T1 scan lasting for 8 min'. The methods state this scan is 6 min 31 sec.

The confusion results from the fact that the T1 scan was recorded during a short practice block that the participants performed inside the scanner. This block lasted 8min during which the 6 min 31 sec T1 scan was recorded. We have made this more clear:

“Once prepared, the participant was placed inside the scanner and performed an 8 min practice block. A T1-weighted scan was acquired during this time in the sagittal orientation using a 3D MPRAGE sequence Brant-Zawadzki et al., (1992) with the following parameters: TR/TI = 2.2/1.1 s, 11° flip angle, FOV 256 x 256 x 180 mm and an 0.8 mm isotropic resolution. Parallel imaging (iPAT = 2) was used to accelerate the acquisition, resulting in an acquisition time of 6 min and 31s.”

(c) Page 10: 'Stimulus presentation' paragraph - 'Stimuli were projected onto a screen behind the subject's head using'. The use of 'subject' should be replaced with 'participant' throughout.

We have corrected the phrasing.

(d) Page 14: Figures 2A and 2B are referred to incorrectly as being in the supplementary material.

We have corrected the mistake.

(e) Figure 5 caption: 'Regressors are build for each time-frequency bin separately.' should be 'built'

We have corrected the mistake.

(f) Page 16, final paragraph: 'Afterwards, each of the resulting regression coefficients (B coefficients) was multiplied with the voxel specific layer weights that have been obtained as described above.' Should be 'were multiplied'

We have corrected the mistake.

(g) Page 17: 'Subsequently, separate analyses were done for two frequency of interest (FOI) ranges centerd around' - typo

We have corrected the mistake.

(h) Page 17 - 'Within these frequency ranges inferential statistics based a cluster level' - missing word. Should be 'based on a cluster level'

We have corrected the mistake.

(i) Page 14 Figure 2B and 5D are referred to incorrectly as being in the supplementary material.

We have corrected the mistake.

(2) fMRI data pre-processing:

Please provide a comment on the EEG-fMRI data quality - e.g. tSNR of EPI data. Perhaps example EPI data could be shown in the supplementary information.

We included the below Figure S11 in Supplementary Figures showing an example EPI. We have also included an illustration of the result of our layering approach. Furthermore, we included a tSNR analysis (see Response to Reviewer 1, point 4).

On a practical note - with 14-minute long runs whilst wearing an EEG cap, I would expect participant motion to be a concern. Could you provide some metrics on perhaps the average of the mean and maximum per subject displacement/rotation?

We ensured that participants receive tactile feedback for their respective head motion from a strip of tape span across their foreheads. This resulted in overall manageable motion during each experimental block. During the main experiment, the average framewise displacement was 0.3 mm, with an average total translation of 1.6 mm and an average total rotation of 1.6 deg within each block.

We have added Figure S13 to Supplementary Figures.

We have added a section to Methods:

“Subject motion per block was low, with a mean (SD) frame-wise displacement Power et al. (2012) of 0.34 mm (0.24 mm) for the main experiment and 0.23 mm (0.22 mm) for the retinotopy (see also Figure S13 in Supplementary Figures).”
